# Normal tissue complication models for clinically relevant acute esophagitis (≥ grade 2) in patients treated with dose differentiated accelerated radiotherapy (DART-bid)

**DOI:** 10.1186/s13014-015-0429-1

**Published:** 2015-05-28

**Authors:** Franz Zehentmayr, Matthias Söhn, Ann-Katrin Exeli, Karl Wurstbauer, Almut Tröller, Heinz Deutschmann, Gerd Fastner, Christoph Fussl, Philipp Steininger, Manfred Kranzinger, Claus Belka, Michael Studnicka, Felix Sedlmayer

**Affiliations:** Univ.-Klinik für Radiotherapie und Radio-Onkologie, Landeskrankenhaus Salzburg, Univ.-Klinikum der Paracelsus Medizinischen Privatuniversität, Müllner Hauptstr. 48, 5020 Salzburg, Austria; Institute for Research and Development of Advanced Radiation Technologies (radART), Paracelsus Medizinische Privatuniversität, Müllner Hauptstr. 48, 5020 Salzburg, Austria; Univ.-Klinik für Pneumologie, Landeskrankenhaus Salzburg, Univ.-Klinikum der Paracelsus Medizinischen Privatuniversität, Müllner Hauptstr. 48, 5020 Salzburg, Austria; Department of Radiotherapy and Radiation Oncology, Ludwig-Maximilians-Universität Munich, Marchioninistr. 15, 81377 Munich, Germany; Department of Radiation Oncology, William Beaumont Health System, 3601 W. Thirteen Mile Road, Royal Oak, MI 48073 USA

**Keywords:** NSCLC (non-small cell lung cancer), Accelerated radiotherapy, Acute esophagitis, NTCP (normal tissue toxicity probability) modeling

## Abstract

**Background:**

One of the primary dose-limiting toxicities during thoracic irradiation is acute esophagitis (AE). The aim of this study is to investigate dosimetric and clinical predictors for AE grade ≥ 2 in patients treated with accelerated radiotherapy for locally advanced non-small cell lung cancer (NSCLC).

**Patients and methods:**

66 NSCLC patients were included in the present analysis: 4 stage II, 44 stage IIIA and 18 stage IIIB. All patients received induction chemotherapy followed by dose differentiated accelerated radiotherapy (DART-bid). Depending on size (mean of three perpendicular diameters) tumors were binned in four dose groups: <2.5 cm 73.8 Gy, 2.5–4.5 cm 79.2 Gy, 4.5–6 cm 84.6 Gy, >6 cm 90 Gy. Patients were treated in 3D target splitting technique. In order to estimate the normal tissue complication probability (NTCP), two Lyman models and the cutoff-logistic regression model were fitted to the data with AE ≥ grade 2 as statistical endpoint. Inter-model comparison was performed with the corrected Akaike information criterion (AIC_c_), which calculates the model’s quality of fit (likelihood value) in relation to its complexity (i.e. number of variables in the model) corrected by the number of patients in the dataset. Toxicity was documented prospectively according to RTOG.

**Results:**

The median follow up was 686 days (range 84–2921 days), 23/66 patients (35 %) experienced AE ≥ grade 2. The actuarial local control rates were 72.6 % and 59.4 % at 2 and 3 years, regional control was 91 % at both time points. The Lyman-MED model (D50 = 32.8 Gy, m = 0.48) and the cutoff dose model (D_c_ = 38 Gy) provide the most efficient fit to the current dataset. On multivariate analysis V38 (volume of the esophagus that receives 38 Gy or above, 95 %-CI 28.2–57.3) was the most significant predictor of AE ≥ grade 2 (HR = 1.05, CI 1.01–1.09, *p* = 0.007).

**Conclusion:**

Following high-dose accelerated radiotherapy the rate of AE ≥ grade 2 is slightly lower than reported for concomitant radio-chemotherapy with the additional benefit of markedly increased loco-regional tumor control. In the current patient cohort the most significant predictor of AE was found to be V38. A second clinically useful parameter in treatment planning may be MED (mean esophageal dose).

**Electronic supplementary material:**

The online version of this article (doi:10.1186/s13014-015-0429-1) contains supplementary material, which is available to authorized users.

## Introduction

Concomitant radiochemotherapy (CRT) is regarded as state-of-the-art treatment for locally advanced non-small cell lung cancer (NSCLC) achieving a median overall survival (OAS) of approximately 17 months in previous reports [1–3] and 28 months in the recent RTOG 0617 study [4]. Similar OAS as in earlier publications was achieved with continuous hyperfractionated accelerated radiotherapy (CHART), but with increased local control [5]. Some studies explored accelerated radiotherapy after induction chemotherapy reporting a median OAS above 20 months [6, 7]. Additionally, loco-regional control can be increased from about 40 % in conventional dose escalation [8, 9] to 70 % [7], yet at the potential cost of increased toxicity.

One of the primary dose limiting toxicities is acute esophagitis (AE), which becomes more frequent with intensified treatment regimens [10, 11], ruling out the benefit of dose escalation by causing unplanned treatment breaks. Consequently, the highest rates of AE are seen in concomitant chemotherapy plus accelerated radiotherapy [10, 12], with severe esophagitis rates (=grade 3) up to 45 % [1]. Several predictors of acute esophagitis (AE) are currently discussed: accelerated radiotherapy, concomitant radiochemotherapy (CRT), mean esophageal dose (MED), Vx (volume receiving at least dose x), Dmax (maximum dose to any point of the esophagus), length of esophagus and proportion of esophageal circumference within a specific isodose, molecular markers [13, 14], and clinical factors such as N-stage, pretreatment dysphagia and body mass index [10, 11, 15–24].

The aim of the present analysis was to elucidate predictors for clinically relevant AE (grade ≥ 2) in a cohort exclusively treated by accelerated radiotherapy after induction chemotherapy.

## Methods

### Patients

Between January 2004 and December 2011, 166 patients with locally advanced NSCLC (stage II–IIIB, UICC/AJCC 7th ed., 2010) were treated with dose differentiated accelerated radiotherapy, twice daily fractions of 1.8 Gy (DART-bid). Patients were discussed in a tumor board together with pneumologists, radiologists, medical oncologists, thoracic surgeons and radiation oncologists. Until 2008, the planning CT included the volume of the tumor and the lymph nodes but did not routinely cover the whole lung. In this study we only analyze the 66 patients with a total lung scan: 4 stage II, 44 stage IIIA and 18 stage IIIB. 38/66 patients were treated within an observational dose escalation study performed between 01/2004 until 12/2009), the remaining 28 were treated along the same treatment protocol but with improved image guidance in a follow up study [7, 25]. Accrual for the 2^nd^ study period terminated in December 2014, however, since the 28 patients from this second cohort had a sufficiently long follow up (mean 637 days), they were included in the present toxicity analysis. Overall, the median age was 65 years (range 44–83 years), the median Karnofsky Performance Score (KPS) was 70 (range 50–90). All studies were carried out with the approval of the responsible ethics committee, in accordance with national law and the Helsinki Declaration of 1975 (in its current, revised form). Informed consent was obtained from all patients.

### Radio-chemotherapy

Patients were irradiated with accelerated radiotherapy (1.8 Gy twice daily) delivered with 15-MV photon beams in 3D-target splitting technique [26]. Depending on tumor size (= mean of three perpendicular diameters) patients were allocated to four prescribed-dose groups: <2.5 cm 73.8 Gy, 2.5–4.5 cm 79.2 Gy, 4.5–6 cm 84.6 Gy, >6 cm 90 Gy. For treatment planning, patients were immobilized in a vacuum cradle in supine position. “Slow CTs” (4 s/slice, thickness 7 mm) were acquired. Due to the prolonged acquisition time the GTV appears enlarged on the CT scan, which means that it is actually a “CTV”. A margin of 7 mm was added to the GTV and positive lymph nodes to generate the PTV as described in a previous report [27]. In the diagnostic work up, a PET-CT was obligatory. Delineation of the tumor PTV was done on the post-chemotherapeutic planning CT in the lung window, the chest window was used for the lymph nodes. For treatment related parameters see Table 1. In the first cohort, image guidance was performed with two orthogonal kV images before each fraction, matching central anatomical structures (esophagus, trachea, main stem bronchi). For the second investigational period (starting in 2010), cone-beam-CT based tumor matching was performed (with or without marker fiducials). The following dose constraints were applied: V20-single-lung <50 %, V25-total-lung <30 %, D_max_ for spinal cord 45 Gy, D_max_ for esophagus 80 Gy.

**Table 1:** Patient and tumor characteristics

Patient and treatment characteristics			*p*-value
Age	Median	65	0,36
	Range	44–83	
KPS	Median	70	0,1
	Range	50–90	
Sex	Male	45	0,13
	Female	21	
Weight loss > 5 %	None	49	0,7
	Yes	17	
Stage	T	T1: 14	<0,001
		T2: 32	
		T3: 11	
		T4:9	
	N	N0: 3	0,07
		N1: 7	
		N2: 43	
		N3: 13	
	UICC	II: 4	x
		IIIA: 44	
		IIIB: 18	
Tumor location	right lung	36	0,8
	left lung	30	
	upper lobe	48	0,44
	middle lobe	2	
	lower lobe	16	
	peripheral	38	0,33
	central	28	
Histology	SCC	39	0,26
	AC	21	
	NOS	6	
Group	I (<2,5 cm)	12	x
	II (2,5–4,5 cm)	32	
	III (4,5–6 cm)	13	
	IV (>6 cm)	9	
PTV tumor (ml)	Median	93	<0,001
	Range	16–528	
Tumor dose (Gy)	Median	79,2	<0,001
	Range	73,8–90	
Lymph node dose (Gy)	Median	61	0,38
	Range	0–90	
ENI^a^ (Gy)	Median	45	0,43
	Range	0–63	

The distribution of patient and tumor characteristics over the four dose groups was tested with the Kruskal-Wallis-Test (x = not tested, ^a^ = elective nodal irradiation). Significant differences were found – as expected – in group-related parameters (T-stage, N-stage, tumor dose, PTV tumor)

Chemotherapy was administered in a sequential mode. All patients received two cycles of platinum based induction chemotherapy before radiotherapy, either with Gemcitabine or Pemetrexed. Antimycotic prophylaxis (Amphotericine B lozengers, 4 times daily) was administered during radiotherapy.

### Assessment of esophagitis

Patients were seen weekly during radiotherapy, 6 weeks after completion of radiotherapy, then quarterly for the first year, every four months during the second and third year, and bi-annually thereafter. AE was documented prospectively, according to RTOG, mild (grade 1), moderate (grade 2), severe (grade 3), life threatening (grade 4), and lethal (grade 5). A patient was scored as having acute esophagitis if the symptoms arose during treatment or within the first 3 months after radiotherapy, thereafter, esophageal toxicity was scored as chronic.

### Esophagus delineation

The external surface of the esophagus was contoured on each slice from the lowest edge of the cricoid cartilage to the gastro-esophageal junction. Oral contrast was administered before acquisition of the planning CT scan.

### DVH parameters

The dose was calculated with collapsed cone (calculation grid: 3 mm) with correction for tissue inhomogeneity (Oncentra Masterplan™ 4.1sp2). For multivariate analysis the following parameters were derived from the dose-volume histogram for each patient: mean esophageal dose (MED), Vx (volume receiving at least dose x) in 2.5 Gy steps from V20 to V70, Dmax, size of the tumor PTV, doses to the tumor, lymph nodes, and elective lymph drainage. A recent review of 18 studies found that the dose range from V20 to V50 including MED was significantly related with AE in most studies [23]. We therefore started our dosimetric analysis at V20. Additionally, the RTOG working group on quantitative analyses of normal tissue effects (QUANTEC) describe a clear trend that V_x_ with x > 40 Gy correlate with AE [28]. NTCP models were calculated for all dose levels between 0 and 91 Gy, which was the highest dose delivered to a single voxel, in 0.1 Gy steps. The volumes for each step are given as percentages.

### Normal tissue complication probability (NTCP)-models

NTCP models assign complication probabilities for organs at risk to given, generally inhomogeneous dose distributions. In this study the clinical endpoint, for which the probability of complication was calculated, was AE ≥ grade 2. We compared three models: (1) The Lyman-EUD model [29–31], which is described by three parameters: the volume-effect parameter *n* (sometimes also denoted as *a = 1/n*) of the equivalent uniform dose (EUD), a dose–response steepness parameter *m* and the dose *EUD*_*50*_ (usually defined as *D*_*50*_) which leads to 50 % probability of complications. (2) The Lyman-MED model, which is a special case of the Lyman-EUD model, with the parameter *n* set to 1. (3) The cutoff dose logistic regression model which correlates the volume *V*_*Dc*_ receiving a dose *D*_*c*_ or higher with retrospective toxicity data in terms of a logistic regression (parameters *β*_*0*_ and *β*_*1*_). For the different NTCP modeling approaches we refer to Söhn et al. [32].

Inter-model comparison was performed with the corrected Akaike information criterion (AIC_c_). The AIC_c_ quantifies the tradeoff between the model’s quality of fit (likelihood value, LL) and its complexity (number of model parameters, k) corrected by the number of cases (i.e. patients) N in the dataset: AIC_c_ = (-2LL + 2 k) + 2*k*(k + 1)/(N-k-1): the smaller the AIC_c_ value, the more efficient the fit of the model to a given dataset [33].

### Biologically equivalent dose

The biologically equivalent dose (EQD_2,T_) was calculated with the linear quadratic formalism including a time factor [34, 35].

### Statistical analysis

The distribution of patient, tumor and treatment characteristics in the dose groups was compared with the non-parametric Kruskal-Wallis-Test. Local and regional control rates as well as OAS were calculated according to the Kaplan-Meier-method. Patients without symptoms (grade 0) or with mild esophagitis (grade 1) were binned in one group, since pre-existing dysphagia is not always easy to differentiate from radiation-induced esophagitis especially in cases with large mediastinal adenopathy. Considering the time to the maximum degree of AE, grade 2 or higher esophagitis was chosen as the statistical endpoint for univariate and multivariate analyses (forward stepwise regression model) to detect potentially predictive factors. We used the software packages Mathematica™ (version 9) for NTCP-modeling and SPSS™ (version 21) for multivariate analyses.

## Results

### Clinical outcome

23/66 (34.8 %) had no esophagitis (grade 0), 20/66 (30.3 %) patients developed mild esophagitis (grade 1). With a median follow up of 686 days (range 84–2921 days), the actuarial local control rates were 72.6 % and 59.4 % at 2 and 3 years, regional control was 91 %. The median overall survival was 25 months (range 19.7–30.3 months) (Additional file 1: Figure S1a-c). No patient was lost to follow up. The median dose to the primary tumor was 79.2 Gy (78 Gy EQD_2,T_; range 73,8–90Gy). Involved lymph nodes were treated with a median dose of 61 Gy (range 0–90 Gy) and elective lymph drainage with a median dose of 45 Gy (0–63 Gy). Patient and tumor related characteristics are summarized in Table 1, treatment related parameters are shown in Table 2.

**Table 2:** Normal tissue complication probability (NTCP) models. This table shows the parameter estimates for the Lyman-EUD-model, the Lyman-MED-model and the cutoff-dose logistic regression model: 95 %-confidence interval, LogLikelihood (LL), corrected Akaike information criterion (AIC_c_)

Model	Parameter estimates	95 % CI		LL	AICc
Lyman-EUD	D50 = 44.9	19.4	75.1	−38.43	83.25
	m = 0.34	0.19	1.06		
	n = 0.34	0.02	19.9		
Lyman-MED	D50 = 32.8	27.7	52.5	−39.08	82.35
	m = 0.48	0.28	1.29		
Cutoff-dose	Dc = 37.9	28.2	57.3	−38.17	82.67
	β0 = -3.06	−5.1	−0.94		
	β1 = 0.06	0.02	0.12		

### Incidences of esophagitis at different toxicity levels

13/66 (19.7 %) cases of esophagitis grade 2 and 10/66 cases (15.2 %) of esophagitis grade 3 were observed. All patients could finish their treatment course without interruption.

### Onset pattern

Esophageal mucosa is a turn-over tissue, hence AE is a result of accumulated dose (Additional file 1: Figure S2). Of those 23 patients who experienced esophagitis ≥ grade 2, 11 (47.8 %) had at least grade 2 esophagitis in week 3 (≥36 Gy), 20/23 patients (87 %) had at least grade 2 esophagitis in week 4 (≥54 Gy). In week 5 (≥72 Gy) the maximum grade of esophagitis was reached in 22/23 (95.7 %) patients, and in week 6 (≥90) all patients had reached the maximum esophagitis grade. In 22/23 (95.7 %) patients with clinically relevant AE, all symptoms had resumed at the 3-months post-radiotherapy control. The time course could not be assessed in one patient with AE grade 3 since he died of pneumonia nine weeks after the end of radiotherapy.

### Chronic toxicity

Two patients experienced chronic esophageal toxicity grade 3. One patient, who had acute esophagitis grade 1, received a stent 7 months after treatment. The second patient, who developed acute esophagitis grade 2, received a stent 10 months after finishing treatment. For the first patient, MED and V38 were 31.5 Gy and 45.2 % respectively, for the second MED and V38 were 25.3 Gy and 34.6 %.

### NTCP-models

**Fig. 1:**
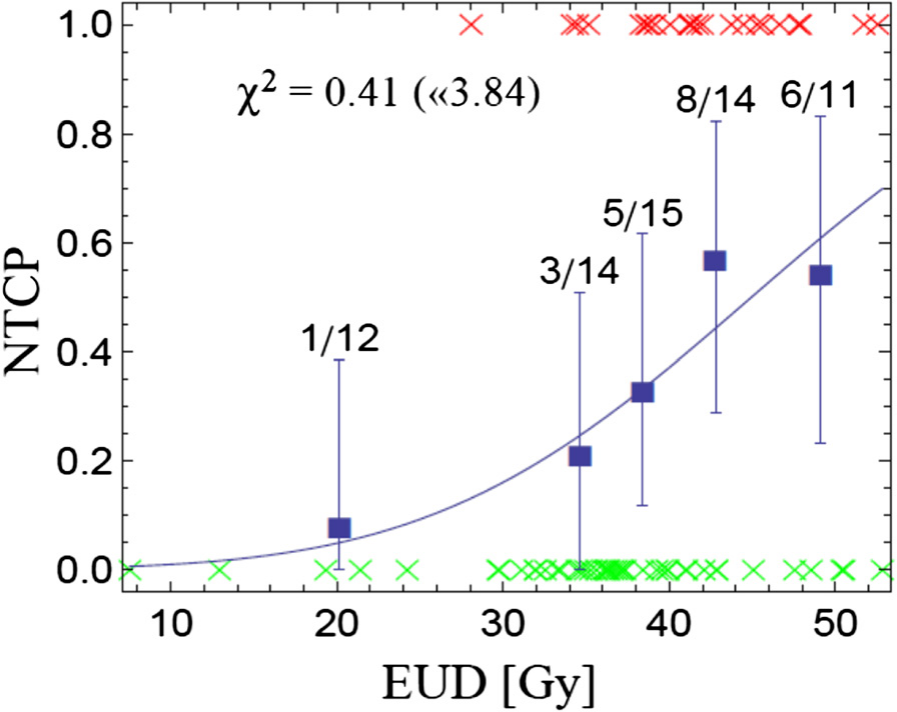
NTCP for AE ≥ grade 2 is shown as a function of equivalent uniform dose (EUD). Red x-symbols represent patients with AE ≥ grade 2, green x-symbols represent patients without toxicity. The actually observed AE rates are shown as bold squares in the centers of corresponding histogram bins (chosen to represent a comparable number of patients). Errors shown are binomial confidence intervals, *χ* ^2^ of the fit and the upper threshold according to Chi-square statistics (α = 5 %) are given for each model

**Fig. 2:**
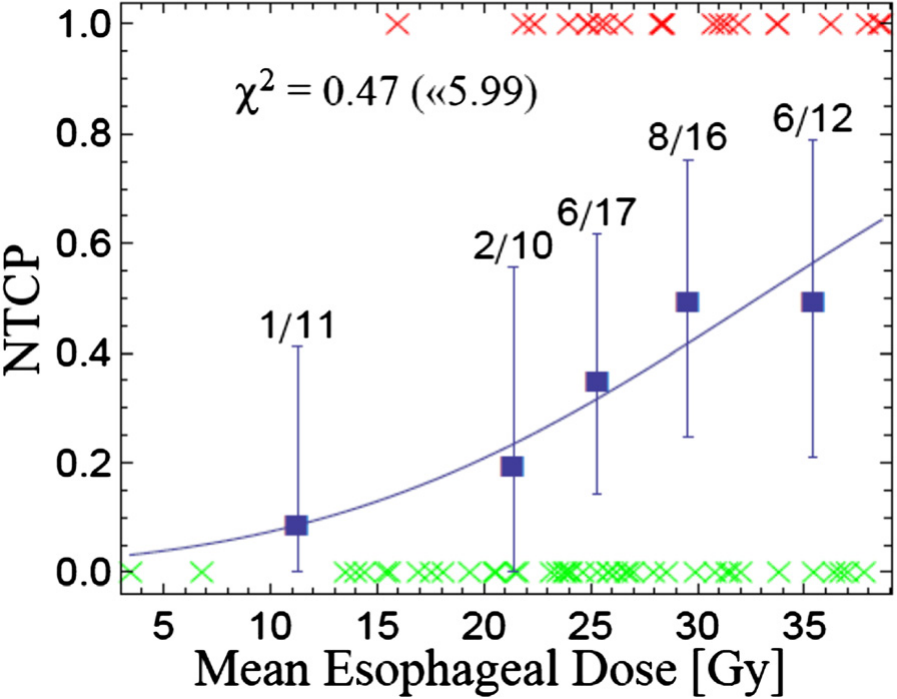
NTCP for AE ≥ grade 2 is shown as a function of mean esophageal dose (MED). Red x-symbols represent patients with AE ≥ grade 2, green x-symbols represent patients without toxicity. The actually observed AE rates are shown as bold squares in the centers of corresponding histogram bins (chosen to represent a comparable number of patients). Errors shown are binomial confidence intervals, *χ* ^2^ of the fit and the upper threshold according to Chi-square statistics (α = 5 %) are given for each model

**Fig. 3:**
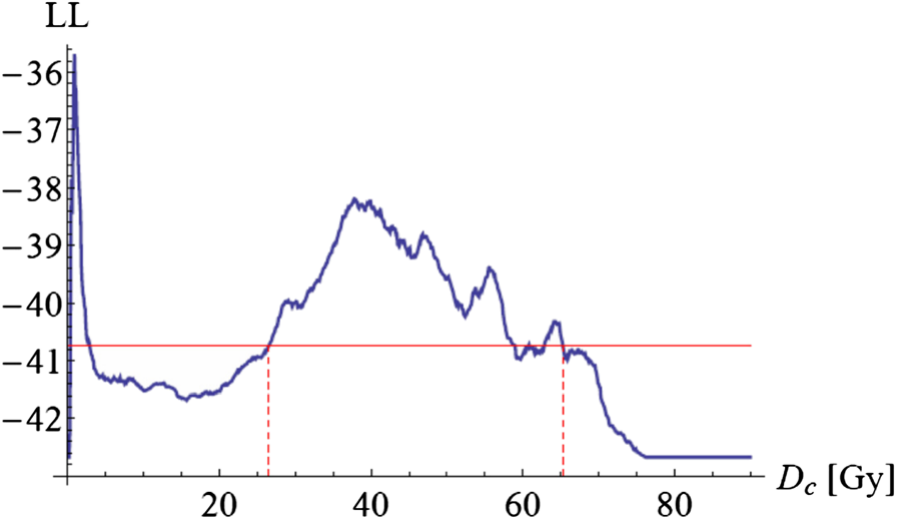
Cutoff dose logistic regression model of AE ≥ grade 2: LogLikelihood (LL) values are plotted against cutoff dose (D_c_); those models with an LL above the horizontal line are statistically significant (*p* < 0.05)

**Fig. 4:**
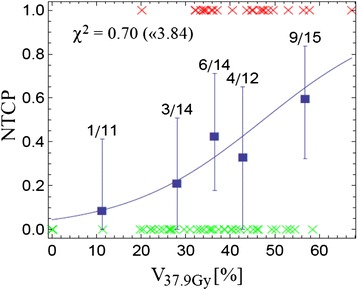
Cutoff dose logistic regression model of AE ≥ grade 2: NTCP based on the cutoff dose model for D_c_ = 37.9 Gy (this D_c_ showed the maximum for LL, see Fig. 3): the probability of AE ≥ grade 2 plotted as a function of the relative volume receiving ≥ 37.9 Gy

We performed two Lyman model fits (Lyman-EUD and Lyman-MED) and a cutoff-dose logistic regression model fit to the data. With an increase in EUD and MED the probability of AE rises (Figs. 1 and 2). In the Lyman-EUD model D50 is 44.9 Gy (95 %-CI: 19.4–75.1), m is 0.34 (95 %-CI: 0.19–1.06), n is 0.34 (95 % CI: 0.02–19.9), in the Lyman-MED model, D_50_ is 32.8 Gy (95 % CI: 27.7–52.5 Gy) and m is 0.48 (95 % CI: 0.28–1.29) (Table 2). The cutoff dose logistic regression model revealed a cutoff dose (D_c_) of 38 Gy (rounded from 37.9 Gy, see Table 2) with the highest predictive potential (Fig. 3) for AE ≥ grade 2. Consistent with the two other NTCP-models, an increase in V38 leads to a higher probability of AE (Fig. 4). The Lyman-MED model and cutoff dose model show the lowest AIC_c_ values (82.35 and 82.67 respectively), therefore building a more efficient model fit to the dataset than the Lyman-EUD model (Table 2).

MED and V38 were calculated for each patient. In the group of patients without or only mild esophagitis, the median MED was 24.0 Gy (range 3.8–36.6 Gy), the group of patients who experienced grade 2 reactions had a median MED of 25.7 Gy (range 16.0–38.4Gy), in grade 3 patients the median MED was 31.2 Gy (24.8–38.4 Gy). In the respective groups the median V38 was 33.5 % (range 0–58.5 %), 37.0 % (range 19.8–57.8 %) and 45.8 % (range 32.3–67.3 %).

### Univariate and multivariate analyses

We hypothesized that the dose given to the esophagus together with selected clinical parameters might be most predictive for AE. Therefore, the following variables were included in the forward stepwise regression (Cox Regression): age, loss of weight, KPS, sex, T, N, tumor location (peripheral versus central), lymph node dose, elective dose, V20 to V70 in 2.5 Gy steps, MED, maximum esophagus dose (D_max_), V38. In the first step (= univariate analysis), tumor location, N-stage, and the V_x_ from V30 to V57.5 and V65 as well as V38 were significant (*p* < 0.05). In the second step (= multivariate model) only V38 retained significance (hazard ratio: 1.05; CI 1.01–1.09, *p* = 0.007).

## Discussion

Although CRT is the standard treatment for locally advanced NSCLC it is feasible only for 30 % of the patient population without dose compromises in chemo- and/or radiotherapy. Especially severe AE would be less well tolerated by those patients with poor functional status [1]. On the other hand DART-bid is feasible for unselected patients [7], including those who would possibly not qualify for full-dose CRT due to co-morbidities. Hence this sequential accelerated approach is a contribution to the treatment of inoperable NSCLC. Despite an increase to >60 % with CRT local control still remains moderate for these patients [4]. It is therefore essential to study the relation of dosimetric parameters and toxicity in the context of dose escalation for improved local control.

Unlike several clinical series reporting about toxicity in patients treated with a variety of modalities within the same treatment series, our patient cohort was exclusively treated with induction chemotherapy followed by dose differentiated accelerated radiotherapy (DART-bid). The median dose was 79.2 Gy (78 Gy EQD_2,T_, twice daily fractions of 1.8 Gy), the incidence of AE ≥ grade 2 is 35 %. NTCP assessment by two Lyman models and the cutoff dose model revealed V38 as the most predictive dosimetric parameter for AE.

The comparison with literature is generally hampered by the use of different statistical endpoints, i.e. either AE grade 2 or 3. With accelerated radiotherapy following induction chemotherapy the reported rate of AE ≥ grade 2 is below 20 %. Patients with smaller tumor burden (lower V20) received higher doses [36]. This concept contrasts with the current study, where patients with larger tumors received higher doses, resulting in a possibly higher rate of AE. With concomitant radio-chemotherapy the reported rate of AE is approximately 40 % [10, 16]. A large range of dosimetric and treatment related parameters is discussed as potentially predictive [10, 11, 15–22, 37, 38]. In a review of 18 studies including 2173 patients, six parameters (V20, V30, V40, V45, V50, MED) were significantly related with AE in at least two thirds of the studies [23]. The RTOG working group on quantitative analyses of normal tissue effects (QUANTEC) uses AE ≥ grade 2 as clinical endpoint, which makes their findings comparable to ours. The authors conclude that there is a clear trend that V_x_ with x > 40 Gy correlate with AE [28].

In the current study we present the Lyman-EUD model parameters for an accelerated radiotherapy scheme. *D*_*50*_ and the volume parameter *n* are smaller than in published cohorts [15, 39]. In the above mentioned study by Belderbos, most patients received sequential radiochemotherapy in standard fractionation, which is less intense than our approach. Patients with overall treatment time (OTT) beyond six weeks were treated in an accelerated mode. The model fit comprises both groups of patients, which may explain the higher *D*_*50*_ than in our analysis. Chapet et al. report on patients with sequential radio-chemotherapy, therefore *D*_*50*_ is even higher [39].

In our patient population, the cutoff-dose model revealed V38 as the most significant predictor: For example, the probability of AE ≥ grade 2 was found to be 30 % or less if V38 was below 34 % (Fig. 4). Since on multivariate analysis, including a range of patient related parameters, the statistically most significant factor for AE prediction was V38, we believe that a specific dose given to the esophagus is more predictive than patient and tumor related parameters. Of note, only a small number of individuals received a relevant percentage of high dose volumes V_x_ with doses > 60 Gy, making it difficult to draw any conclusions from our data in these dose ranges.

It is important to note that the peak around D_c_ = 38 Gy is not very sharply defined in Fig. 3, indicating that V_x_ with doses in the same range (e.g. V35 to V45) show correlations to toxicity with similar significance. It remains unclear if this finding has an actual radiobiological background, or if it is rather an effect of the specific treatment technique (beam arrangement etc.) which induces correlations in the DVH dose bins. Statistical methods like principal component analysis for DVHs to elucidate such effects have been proposed in literature [40]. A second – clinically useful – dosimetric parameter, which is possibly less affected by different beam arrangements, might be MED. The Lyman-MED model also describes the dataset efficiently. Thus, based on the findings for our patient cohort, if we accept – again – a 30 %-probability of AE ≥ grade 2, the MED should not exceed 24 Gy in patients treated with DART-bid (Fig. 2). Huang et al. also propose an MED model based on AE ≥ grade 2 to estimate NTCP [37]. Judging from the logistic regression graph in their paper, in order to achieve a 30 %-probability of AE ≥ grade 2, MED should be < 27 Gy in patients treated with radiotherapy only or sequential radio-chemotherapy. This is slightly higher than in our model and possibly due to the fact that the current study included only patients who received accelerated radiotherapy.

Because esophageal mucosa is a turn-over tissue, AE depends on accumulated total dose. In the study by Wei, which included patients with concomitant radio-chemotherapy only, AE ≥ grade 2 started during the second week of radiotherapy with increasing incidences towards the end of the treatment series [21]. This onset pattern is comparable to DART-bid.

Still, one has to bear in mind that current models do not account for the velocity of dose accumulation in accelerated schedules. On top of that, our model fit as well as those presented in other studies are descriptive of the current dataset, and an extrapolation to another irradiation technique, e.g. IMRT, has to be taken with caution. In addition, the assessment of AE is physician dependent and can be blurred by esophageal infection, pre-existing gastro-esophageal reflux and/or dysphagia [28], which is a major limitation for a comparison between studies. Finally, the delineation of the esophagus on the planning CT scan is crucial: Chapet excluded the cervical esophagus in his analysis [39], whereas in the current analysis – like Belderbos [15] – we delineated the esophagus to the lower limit of the cricoid cartilage. Additionally, the daily dose exposure of the esophagus may vary due to inter- and intrafraction organ mobility [41].

## Conclusion

The rate of AE ≥ grade 2 in DART-bid is slightly lower than in standard concomitant radio-chemotherapy schedules, despite higher total doses and hence, higher loco-regional tumor control rates. The most significant predictor of AE was found to be V38 (volume of the esophagus that receives at least 38 Gy, 95 %-CI 28.2–57.3). Although the results of the current study need to be validated in independent cohorts treated similarly, our findings allow us to assume that the probability of AE ≥ grade 2 is 30 % or less if V38 does not exceed 34 %. A second clinically useful parameter in treatment planning may be MED (mean esophageal dose).

## Additional file

Additional file 1: Figure S1. Clinical outcome. With a median follow up of 686 days, the actuarial LC rates for all patients (n = 66) are 73 % and 59 % at 2 and 3 years, regional control is 91 %, median OAS in 25 months. **Figure S2** Onset pattern and time course of acute esophagitis (AE) ≥ grade 2. Cumulative incidence of AE (66 patients = 100 %): < grade 2 (blue), grade 2 (red) and grade 3 (green). AE ≥ grade 2 starts in week 3 (11 patients), increases towards the last week of treatment (23 cases of AE ≥ grade 2) and resumes completely in 22 patients within 12 weeks after the end of radiotherapy (exact numbers in the table below the graph, * 1 patient died for pneumonia 9 weeks after completion of radiotherapy). **Table S1** Univariate and multivariate analyses of dosimetric and clinically relevant parameters (*Cox Regression, forward stepwise): KPS Karnofsky Performance Score, MED mean esophageal dose, D_max_ maximum dose to the esophagus; significant p-values (<0.05) in bold letters. On multivariate analysis only V38 retained significance (HR: 1.05; CI 1.01–1.09, *p* = 0.007).

## Abbreviations

AE: Acute esophagitis
DART-bid: Dose differentiated accelerated radiotherapy
NTCP: Normal tissue toxicity complication probability
MED: Mean esophageal dose
D_c_: Cutoff dose
NSCLC: Non-small cell lung cancer
CRT: Concomitant radiochemotherapy
OAS: Overall survival
CHART: Continuous hyperfractionated accelerated radiotherapy
LC: Local control
Vx: Volume receiving a certain dose
Dmax: Maximum dose to any point of an organ
KPS: Karnofsky Performance Score
EUD: Equivalent uniform dose
n: Volume-effect parameter in the Lyman-EUD-model
m: Dose–response steepness parameter in the Lyman-EUD-model
EUD_50_ (= D_50_): Dose that leads to 50 % probability of complications
AIC_c_: Corrected Akaike information criterion
EQD_2,T_: Time factor corrected biologically equivalent dose
CI: Confidence interval
QUANTEC: Quantitative analyses of normal tissue effects
OTT: Overall treatment time
DVH: Dose volume histogram
IMRT: Intensity modulated radiotherapy

## References

[1] Curran WJ, Paulus R, Langer CJ, Komaki R, Lee JS, Hauser S (2011). Sequential vs. concurrent chemoradiation for stage III non-small cell lung cancer: randomized phase III trial RTOG 9410. J Natl Cancer Inst.

[2] Furuse K, Fukuoka M, Kawahara M, Nishikawa H, Takada Y, Kudoh S (1999). Phase III study of concurrent versus sequential thoracic radiotherapy in combination with mitomycin, vindesine, and cisplatin in unresectable stage III non-small-cell lung cancer. J Clin Oncol.

[3] Fournel P, Robinet G, Thomas P, Souquet PJ, Lena H, Vergnenegre A (2005). Randomized phase III trial of sequential chemoradiotherapy compared with concurrent chemoradiotherapy in locally advanced non-small-cell lung cancer: Groupe Lyon-Saint-Etienne d’Oncologie Thoracique-Groupe Francais de Pneumo-Cancerologie NPC 95-01 Study. J Clin Oncol.

[4] Bradley JD, Paulus R, Komaki R, Masters G, Blumenschein G, Schild S (2015). Standard-dose versus high-dose conformal radiotherapy with concurrent and consolidation carboplatin plus paclitaxel with or without cetuximab for patients with stage IIIA or IIIB non-small-cell lung cancer (RTOG 0617): a randomised, two-by-two factorial phase 3 study. Lancet Oncology.

[5] Saunders M, Dische S, Barrett A, Harvey A, Griffiths G, Palmar M (1999). Continuous, hyperfractionated, accelerated radiotherapy (CHART) versus conventional radiotherapy in non-small cell lung cancer: mature data from the randomised multicentre trial. CHART Steering committee. Radiother Oncol.

[6] van Baardwijk A, Wanders S, Boersma L, Borger J, Ollers M, Dingemans AM (2010). Mature results of an individualized radiation dose prescription study based on normal tissue constraints in stages I to III non-small-cell lung cancer. J Clin Oncol.

[7] Wurstbauer K, Deutschmann H, Dagn K, Kopp P, Zehentmayr F, Lamprecht B (2013). DART-bid (Dose-differentiated accelerated radiation therapy, 1.8 Gy twice daily)–a novel approach for non-resected NSCLC: final results of a prospective study, correlating radiation dose to tumor volume. Radiat Oncol..

[8] Kong FM, Ten Haken RK, Schipper MJ, Sullivan MA, Chen M, Lopez C (2005). High-dose radiation improved local tumor control and overall survival in patients with inoperable/unresectable non-small-cell lung cancer: long-term results of a radiation dose escalation study. Int J Radiat Oncol Biol Phys.

[9] Wurstbauer K, Weise H, Deutschmann H, Kopp P, Merz F, Studnicka M (2010). Non-small cell lung cancer in stages I-IIIB: long-term results of definitive radiotherapy with doses >/= 80 Gy in standard fractionation. Strahlenther Onkol.

[10] Werner-Wasik M, Pequignot E, Leeper D, Hauck W, Curran W (2000). Predictors of severe esophagitis include use of concurrent chemotherapy, but not the length of irradiated esophagus: a multivariate analysis of patients with lung cancer treated with nonoperative therapy. Int J Radiat Oncol Biol Phys.

[11] Ahn SJ, Kahn D, Zhou S, Yu X, Hollis D, Shafman TD (2005). Dosimetric and clinical predictors for radiation-induced esophageal injury. Int J Radiat Oncol Biol Phys.

[12] Mauguen A, Le Pechoux C, Saunders MI, Schild SE, Turrisi AT, Baumann M (2012). Hyperfractionated or accelerated radiotherapy in lung cancer: an individual patient data meta-analysis. J Clin Oncol.

[13] Guerra JL, Gomez D, Wei Q, Liu Z, Wang LE, Yuan X (2012). Association between single nucleotide polymorphisms of the transforming growth factor beta1 gene and the risk of severe radiation esophagitis in patients with lung cancer. Radiother Oncol.

[14] Dikomey E (2013). Predictive marker for acute normal tissue toxity in radiotherapy of non-small cell lung cancer. Strahlenther Onkol.

[15] Belderbos J, Heemsbergen W, Hoogeman M, Pengel K, Rossi M, Lebesque J (2005). Acute esophageal toxicity in non-small cell lung cancer patients after high dose conformal radiotherapy. Radiother Oncol.

[16] Patel AB, Edelman MJ, Kwok Y, Krasna MJ, Suntharalingam M (2004). Predictors of acute esophagitis in patients with non-small-cell lung carcinoma treated with concurrent chemotherapy and hyperfractionated radiotherapy followed by surgery. Int J Radiat Oncol Biol Phys.

[17] Bradley J, Deasy JO, Bentzen S, El-Naqa I (2004). Dosimetric correlates for acute esophagitis in patients treated with radiotherapy for lung carcinoma. Int J Radiat Oncol Biol Phys.

[18] Qiao WB, Zhao YH, Zhao YB, Wang RZ (2005). Clinical and dosimetric factors of radiation-induced esophageal injury: radiation-induced esophageal toxicity. World J Gastroenterol.

[19] Singh AK, Lockett MA, Bradley JD (2003). Predictors of radiation-induced esophageal toxicity in patients with non-small-cell lung cancer treated with three-dimensional conformal radiotherapy. Int J Radiat Oncol Biol Phys.

[20] Kim TH, Cho KH, Pyo HR, Lee JS, Han JY, Zo JI (2005). Dose-volumetric parameters of acute esophageal toxicity in patients with lung cancer treated with three-dimensional conformal radiotherapy. Int J Radiat Oncol Biol Phys.

[21] Wei X, Liu HH, Tucker SL, Liao Z, Hu C, Mohan R (2006). Risk factors for acute esophagitis in non-small-cell lung cancer patients treated with concurrent chemotherapy and three-dimensional conformal radiotherapy. Int J Radiat Oncol Biol Phys.

[22] Takeda K, Nemoto K, Saito H, Ogawa Y, Takai Y, Yamada S (2005). Dosimetric correlations of acute esophagitis in lung cancer patients treated with radiotherapy. Int J Radiat Oncol Biol Phys.

[23] Rose J, Rodrigues G, Yaremko B, Lock M, D’Souza D (2009). Systematic review of dose-volume parameters in the prediction of esophagitis in thoracic radiotherapy. Radiother Oncol.

[24] Manapov F, Sepe S, Niyazi M, Belka C, Friedel G, Budach W (2013). Dose-volumetric parameters and prediction of severe acute esophagitis in patients with locally-advanced non small-cell lung cancer treated with neoadjuvant concurrent hyperfractionated-accelerated chemoradiotherapy. Radiat Oncol..

[25] Zehentmayr F, Wurstbauer K, Deutschmann H, Fussl C, Kopp P, Dagn K et al. DART-bid: dose-differentiated accelerated radiation therapy, 1.8 Gy twice daily: high local control in early stage (I/II) non-small-cell lung cancer. Strahlenther Onkol. 2014. doi: 10.1007/s00066-014-0754-6. 10.1007/s00066-014-0754-6 25245469

[26] Wurstbauer K, Deutschmann H, Kopp P, Merz F, Scholler H, Sedlmayer F (2009). Target splitting in radiation therapy for lung cancer: further developments and exemplary treatment plans. Radiat Oncol..

[27] Wurstbauer K, Deutschmann H, Kopp P, Sedlmayer F (2005). Radiotherapy planning for lung cancer: slow CTs allow the drawing of tighter margins. Radiother Oncol.

[28] Werner-Wasik M, Yorke E, Deasy J, Nam J, Marks LB (2010). Radiation dose-volume effects in the esophagus. Int J Radiat Oncol Biol Phys.

[29] Lyman JT (1985). Complication probability as assessed from dose-volume histograms. Rad Res Suppl.

[30] Burman C, Kutcher GJ, Emami B, Goitein M (1991). Fitting of normal tissue tolerance data to an analytic function. Int J Radiat Oncol Biol Phys.

[31] Kutcher GJ, Burman C (1989). Calculation of complication probability factors for non-uniform normal tissue irradiation: the effective volume method. Int J Radiat Oncol Biol Phys.

[32] Söhn M, Yan D, Liang J, Meldolesi E, Vargas C, Alber M (2007). Incidence of late rectal bleeding in high-dose conformal radiotherapy of prostate cancer using equivalent uniform dose-based and dose-volume-based normal tissue complication probability models. Int J Radiat Oncol Biol Phys.

[33] Hurvich CM, Tsai CL (1989). Regression and time-series model selection in small samples. Biometrika.

[34] van Baardwijk A, Reymen B, Wanders S, Borger J, Ollers M, Dingemans AM (2012). Mature results of a phase II trial on individualised accelerated radiotherapy based on normal tissue constraints in concurrent chemo-radiation for stage III non-small cell lung cancer. Eur J Cancer.

[35] Fowler JF, Tome WA, Fenwick JD, Mehta MP (2004). A challenge to traditional radiation oncology. Int J Radiat Oncol Biol Phys.

[36] Bradley J, Graham MV, Winter K, Purdy JA, Komaki R, Roa WH (2005). Toxicity and outcome results of RTOG 9311: a phase I-II dose-escalation study using three-dimensional conformal radiotherapy in patients with inoperable non-small-cell lung carcinoma. Int J Radiat Oncol Biol Phys.

[37] Huang EX, Bradley JD, El Naqa I, Hope AJ, Lindsay PE, Bosch WR (2012). Modeling the risk of radiation-induced acute esophagitis for combined Washington University and RTOG trial 93–11 lung cancer patients. Int J Radiat Oncol Biol Phys.

[38] Rodriguez N, Algara M, Foro P, Lacruz M, Reig A, Membrive I (2009). Predictors of acute esophagitis in lung cancer patients treated with concurrent three-dimensional conformal radiotherapy and chemotherapy. Int J Radiat Oncol Biol Phys.

[39] Chapet O, Kong FM, Lee JS, Hayman JA, Ten Haken RK (2005). Normal tissue complication probability modeling for acute esophagitis in patients treated with conformal radiation therapy for non-small cell lung cancer. Radiother Oncol.

[40] Söhn M, Alber M, Yan D (2007). Principal component analysis-based pattern analysis of dose-volume histograms and influence on rectal toxicity. Int J Radiat Oncol Biol Phys.

[41] Dieleman EM, Senan S, Vincent A, Lagerwaard FJ, Slotman BJ (2007). Four-dimensional computed tomographic analysis of esophageal mobility during normal respiration. Int J Radiat Oncol Biol Phys.

