# Supplementary material for: Normal tissue complication models for clinically relevant acute esophagitis (≥ grade 2) in patients treated with dose differentiated accelerated radiotherapy (DART-bid)
Source: Radiat Oncol. 2015 May 28;10:121. doi: 10.1186/s13014-015-0429-1 (PMC4450607; doi:10.1186/s13014-015-0429-1)
Supplement: Additional file 1: Figure S1. — Clinical outcome. With a median follow up of 686 days, the actuarial LC rates for all patients (n = 66) are 73 % and 59 % at 2 and 3 years, regional control is 91 %, median OAS in 25 months. Figure S2 Onset pattern and time course of acute esophagitis (AE) ≥ grade 2. Cumulative incidence of AE (66 patients = 100 %): < grade 2 (blue), grade 2 (red) and grade 3 (green). AE ≥ grade 2 starts in week 3 (11 patients), increases towards the last week of treatment (23 cases of AE ≥ grade 2) and resumes completely in 22 patients within 12 weeks after the end of radiotherapy (exact numbers in the table below the graph, * 1 patient died for pneumonia 9 weeks after completion of radiotherapy). Table S1 Univariate and multivariate analyses of dosimetric and clinically relevant parameters (*Cox Regression, forward stepwise): KPS Karnofsky Performance Score, MED mean esophageal dose, Dmax maximum dose to the esophagus; significant p-values (<0.05) in bold letters. On multivariate analysis only V38 retained significance (HR: 1.05; CI 1.01–1.09, p = 0.007). [file 13014_2015_429_MOESM1_ESM.docx]

**Figure 1a – 1c: Clinical outcome**

With a median follow up of 686 days, the actuarial LC rates for all patients (n = 66) are 73% and 59% at 2 and 3 years, regional control is 91%, median OAS in 25 months.

Fig. 1a


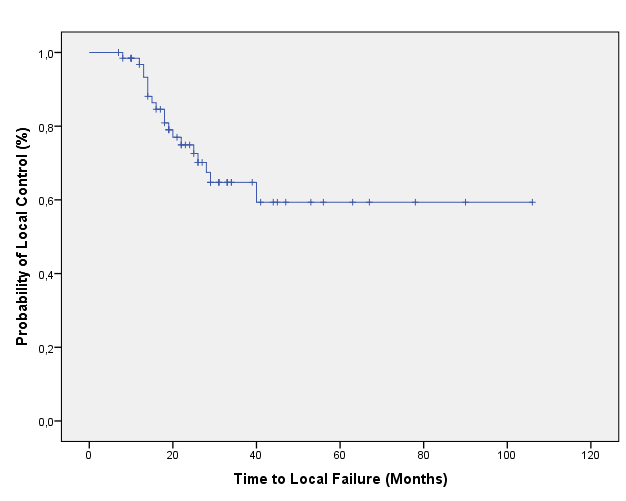


Fig. 1b


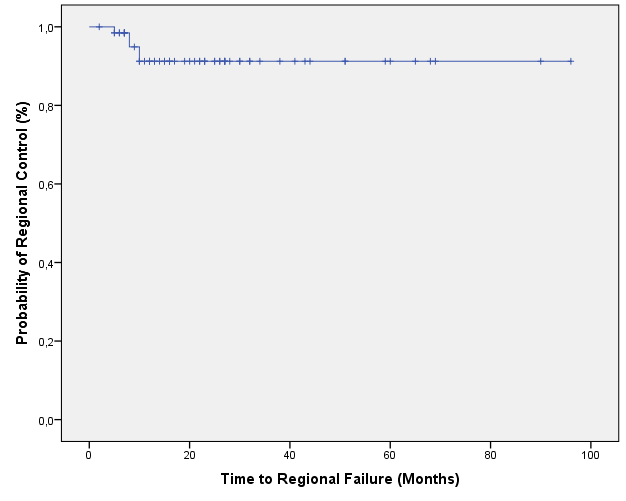


Fig. 1c


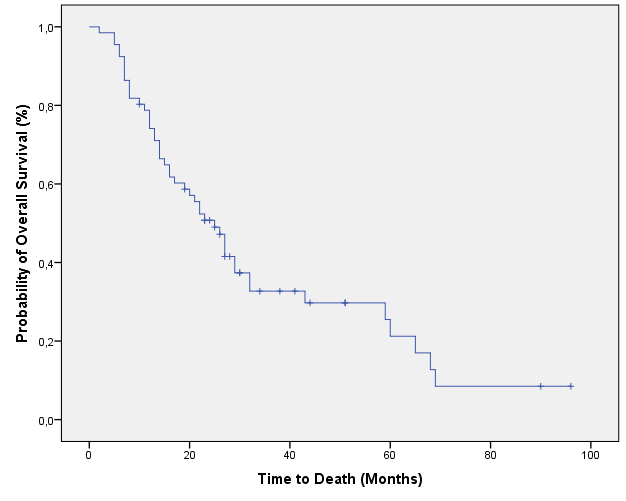


**Fig. 2 Onset pattern and time course of acute esophagitis (AE) ≥ grade 2**

Cumulative incidence of AE (66 patients = 100%): < grade 2 (blue), grade 2 (red) and grade 3 (green). AE ≥ grade 2 starts in week 3 (11 patients), increases towards the last week of treatment (23 cases of AE ≥ grade 2) and resumes completely in 22 patients within 12 weeks after the end of radiotherapy (exact numbers in the table below the graph, * 1 patient died for pneumonia 9 weeks after completion of radiotherapy).

| AE grade | w 1 | w 2 | w 3 | w 4 | w 5 | w 6 | 6 w after RT* | 12 w after RT* |
| --- | --- | --- | --- | --- | --- | --- | --- | --- |
| 0° / 1° | 66 | 66 | 55 | 46 | 44 | 43 | 20 | 22 |
| 2° | 0 | 0 | 10 | 14 | 14 | 13 | 1 | 0 |
| 3° | 0 | 0 | 1 | 6 | 8 | 10 | 1 | 0 |

Table 1 Univariate and multivariate analyses of dosimetric and clinically relevant parameters

(*Cox Regression, forward stepwise): KPS Karnofsky Performance Score, MED mean esophageal dose, D_max_ maximum dose to the esophagus; significant p-values (< 0.05) in bold letters. On multivariate analysis only V38 retained significance (HR: 1.05; CI 1.01 – 1.09, p = 0.007).

| Dosimetric parameter | p-value* | |
| --- | --- | --- |
|  | univariate | multivariate |
| Age | 0.301 | 0.644 |
| Loss of weight | 0.422 | 0.858 |
| Sex | 0.156 | 0.473 |
| KPS | 0.694 | 0.323 |
| T | 0.924 | 0.687 |
| N | **0.012** | 0.139 |
| Location (peripheral versus central) | **0.026** | 0.051 |
| Lymph node dose | 0.651 | 0.759 |
| Elective lymph node dose | 0.815 | 0.160 |
| MED | 0.009 | 0.657 |
| Dmax | 0.130 | 0.650 |
| V20 | 0.148 | 0.528 |
| V22.5 | 0.102 | 0.578 |
| V25 | 0.082 | 0.555 |
| V27.5 | 0.051 | 0.685 |
| V30 | **0.035** | 0.653 |
| V32.5 | **0.025** | 0.567 |
| V35 | **0.015** | 0.567 |
| V37.5 | **0.008** | 0.386 |
| V38 | **0.007** | **0.007** |
| V40 | **0.007** | 0.668 |
| V42.5 | **0.011** | 0.543 |
| V45 | **0.015** | 0.683 |
| V47.5 | **0.011** | 0.941 |
| V50 | **0.017** | 0.993 |
| V52.5 | **0.034** | 0.941 |
| V55 | **0.019** | 0.640 |
| V57.5 | **0.034** | 0.636 |
| V60 | 0.062 | 0.810 |
| V62.5 | 0.052 | 0.710 |
| V65 | **0.041** | 0.465 |
| V67.5 | 0.063 | 0.483 |
| V70 | 0.148 | 0.639 |
